# Analysis of Dental Enamel Remineralization: A Systematic Review of Technique Comparisons

**DOI:** 10.3390/bioengineering10040472

**Published:** 2023-04-12

**Authors:** Giuseppina Malcangi, Assunta Patano, Roberta Morolla, Matteo De Santis, Fabio Piras, Vito Settanni, Antonio Mancini, Daniela Di Venere, Francesco Inchingolo, Alessio Danilo Inchingolo, Gianna Dipalma, Angelo Michele Inchingolo

**Affiliations:** Department of Interdisciplinary Medicine, University of Bari “Aldo Moro”, 70124 Bari, Italy; giuseppinamalcangi@libero.it (G.M.); assuntapatano@gmail.com (A.P.); robertamorolla@gmail.com (R.M.); m.desantis51@studenti.uniba.it (M.D.S.); dott.fabio.piras@gmail.com (F.P.); v.settanni@libero.it (V.S.); dr.antonio.mancini@gmail.com (A.M.); daniela.divenere@uniba.it (D.D.V.); giannadipalma@tiscali.it (G.D.); angeloinchingolo@gmail.com (A.M.I.)

**Keywords:** remineralization, enamel, randomized controlled trial, tooth demineralization, systematic review, white spot lesion, enamel decalcification, fluoride, MI paste plus

## Abstract

The demineralization process conditions the structure of the enamel and begins with a superficial decalcification procedure that makes the enamel surface porous and gives it a chalky appearance. White spot lesions (WSLs) are the first clinical sign that can be appreciated before caries evolves into cavitated lesions. The years of research have led to the testing of several remineralization techniques. This study’s objective is to investigate and assess the various methods for remineralizing enamel. The dental enamel remineralization techniques have been evaluated. A literature search on PubMed, Scopus, and Web of Science was performed. After screening, identification, and eligibility processes 17 papers were selected for the qualitative analysis. This systematic review identified several materials that, whether used singly or in combination, can be effective in the process of remineralizing enamel. All methods have a potential for remineralization when they come into contact with tooth enamel surfaces that have early-stage caries (white spot lesions). From the studies conducted in the test, all of the substances used to which fluoride has been added contribute to remineralization. It is believed that by developing and researching new remineralization techniques, this process might develop even more successfully.

## 1. Introduction

The process of demineralization is a gradual process that leads to the gradual dissolution of hydroxyapatite crystals in enamel, resulting in the loss of tooth tissue. This process will be discussed extensively below, along with its causes. Demineralization of enamel and dentin appears to be an increasingly common problem, despite technological development in oral hygiene and awareness campaigns. The main consequences related to it are increased dentin sensitivity and the development of dental caries [1,2,3].

Dental enamel is an acellular, hard, avascular tissue, consisting of 96% inorganic material (hydroxyapatite nanocrystals), 3% water, and 1% organic component [4,5].

The enamel protein matrix is produced by ameloblasts and later mineralized through calcium phosphate crystals [6,7]. Enamel is an acellular tissue in that ameloblasts die soon after producing it [8].

Its highly mineralized structure makes it on the one hand very resistant, and on the other hand susceptible to the phenomenon of demineralization [2,6].

Enamel does not possess a precise color, but has a fundamental property called translucency. It is a typical characteristic of enamel and is closely associated with the structure of dental enamel itself. Specifically, translucency is an optical effect that is closely dependent on the spaces between hydroxyapatite crystals. In the early stages, to clinically accurately identify carious pathology, it was necessary to dry the enamel surface [9].

With the progression of carious pathology, however, the spaces between enamel crystals widen. This change results in the formation of a chalky-looking lesion even without drying of the surface, which appears distinguishable from the surrounding healthy enamel [10]. Translucency is lost when the dehydration of dental enamel begins. This occurs as a result of the gradual replacement of water by air between the spaces present between enamel prisms [9].

Translucence can be defined quantitatively by the enamel refractive index (ERI). Specifically, the refractive index is the ratio of the speed of light in a vacuum to the speed of light in the medium traversed [11]. The denser the material through which light passes, the slower the passage and propagation of light through that body. Dental enamel has a refractive index of 1.62. Because water has a refractive index of 1.33 and air has a refractive index of one, greater scattering occurs at the enamel/air interface [12].

The main functions of enamel are to protect dentin and pulp from thermal shock, mechanical stress, chemical corrosion, and bacterial infection [13,14]. One of the main causes of the occurrence of carious lesions resulting in the loss of tooth structure is attributable to the presence of bacteria.

Different types of microorganisms constitute the biofilm that adheres to the tooth surface [15]. This microbial colony stabilizes the oral pH and protects the environment from exogenous and pathogenic species [16,17,18].

**Figure 1:**
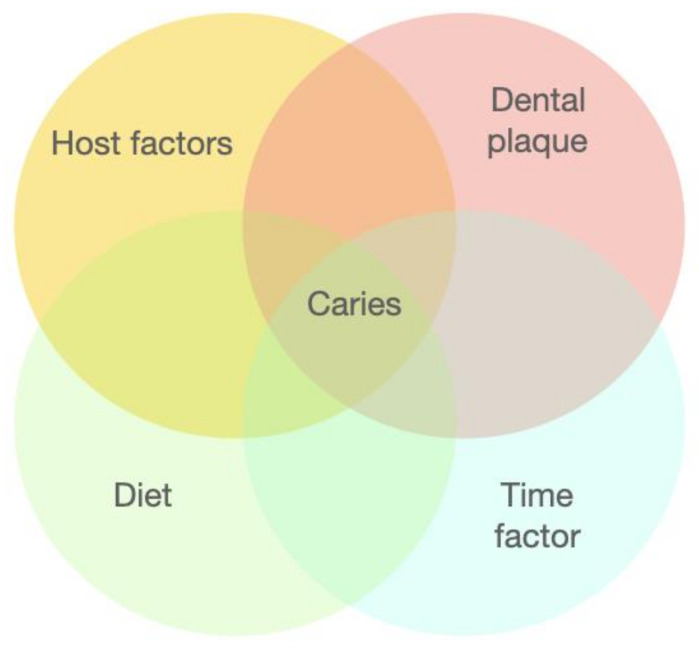
Overview diagram on factors contributing to the formation of the carious process.

Disruption of this balance is the result of the complex interaction between hard tissue, biofilm, and metabolic processes. Fluoride’s influence on the structure and stability of tooth minerals, as well as its absorption into the developing mineral, has been the focus of much research in dentistry due to this ion’s well-documented cariostatic action. Among the endogenous causes attributable to tooth surface demineralization is gastroesophageal reflux, while an exogenous cause is a diet high in sugar, especially if it is not followed by proper oral hygiene. [19,20]. Cariogenic sugars are introduced into the oral cavity. A diet rich in sugars favors cariogenic bacterial metabolism, which becomes the predominant strain in the biofilm that covers the oral surfaces. The latter, settling on the tooth surface, damages it. If this process is not stopped or limited it leads to the occurrence of cavitated lesions (Figure 1).

These cariogenic bacteria can withstand low pH environments, transform monosaccharides into acidic substances, and produce extracellular polysaccharides that allow and make it easier for the bacteria to adhere to plaque. Within the oral microbiome, there is a dynamic balance between the host and the oral flora. Pathology occurs in the case of an alteration of this balance in favor of cariogenic bacteria [21].

In a physiological balance of saliva, a normal pH is between 6.5 and 7.4, in which it is supersaturated with calcium and phosphate ions, so demineralization does not take place. Bacterial acids (such as Streptococcus mutans) and acids from food or drink tend to lower the pH and determine mineral loss [2,22].

Specifically, when the oral pH falls below the value of 5.5, the concentration of phosphate ions in saliva decreases, and hydroxyapatite (HA) crystals begin to dissolve [23,24].

Saliva, therefore, has an important buffering capacity and acts as a transport vehicle for ions, such as fluoride, that can be incorporated into tissues [25].

**Figure 2:**
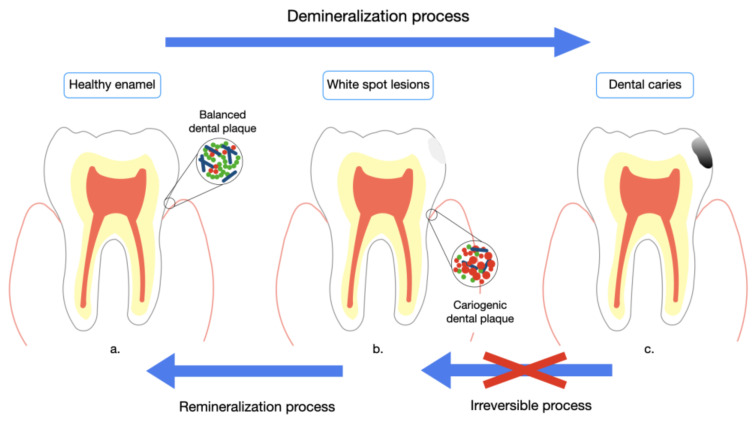
Demineralization and remineralization processes. (**a**) Enamel is healthy with balanced bacterial plaque. (**b**) The plaque changes and becomes cariogenic and starts the formation of a WSL (reversible lesion). (**c**) If the WSL is left untreated and the bacterial plaque is not removed, an irreversible carious process begins (cavitated lesion).

Among the most prominent clinical signs preceding the onset of caries are WSLs, which are the first clinical sign of dental demineralization. On objective examination, they appear as opaque, chalky surfaces as a result of increased enamel porosity [26,27]. If caught early, WSLs result in reversible lesions. If, on the contrary, the acidic environment is not eliminated or reduced, WSLs can evolve into cavitated lesions (Figure 2) [18,28,29].

Loss of mineralized structure beneath an apparently intact layer characterizes carious lesions. There is an increase in porosity within the lesion itself, which gives rise to the chalky appearance of white spots [10].

WSLs can be classified into active lesions if they have a chalky, rough appearance and inactive lesions if they have a shiny, smooth appearance [30].

Very often, WSLs can be visible both clinically and radiographically even after the use of remineralization agents [31]. This happens because the deepest part of the lesion has the lowest potential for remineralization and continues to diffuse light differently. Therefore, even if the white spot becomes hard, smooth, and shiny (and therefore inactive), some internal opacity is likely to remain [32].

To try minimize the occurrence of carious lesions, it is essential to implement effective prevention protocols.

Regenerative dentistry is a revolutionary idea that pushes mainstream dentistry to accelerate dental research and integrate scientific discoveries into innovative treatments in the future. The approach is founded on an understanding of the underlying mechanisms of tooth development, as well as the biological processes of healing and repair, resulting in a solid understanding of principles that could be applied in maximizing the inherent healing potential of dental tissues or regenerating (engineering) the damaged tissue enamel.

**Figure 3:**
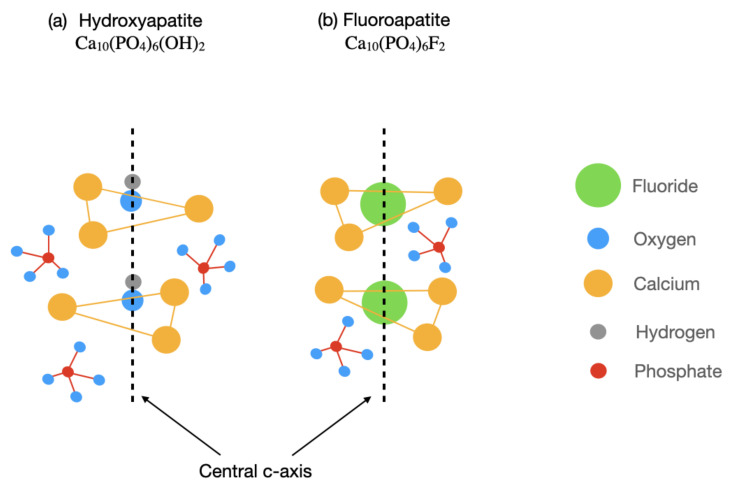
(**a**) Structure of hydroxyapatite: the hydroxyl ion located in the central c-axis is surrounded by calcium and phosphates and by a hexagonal of calcium. (**b**) Representation of the remineralization process: there is replacement of the hydroxyl ion by a fluoride ion in the center of the molecule.

Remineralization from an external source allows for the deposition of new minerals within the tooth structure [33]. The altered mineral has a greater uptake of locally applied fluoride, which allows for more effective remineralization of non-cavitated lesions. This mechanism can be better understood by looking at the structure of hydroxyapatite (Figure 3) [34,35,36].

The crystal structure of hydroxyapatite exhibits a planar–hexagonal nature of calcium and phosphate ions surrounding the central hydroxyl c-axis [37].

During remineralization, fluoride is classically incorporated by replacing or displacing hydroxyl ions.

As a result of fluoride incorporation, Ca–F covalent bonds and/or OH–F hydrogen bonds are formed, and the unit cell volume of the apatite is reduced, increasing the hardness and acid resistance (chemical durability) of dental enamel (Figure 3b) [38,39,40,41].

Another important function of fluoride ions is to act as antibacterial agents and as inhibitors of the bacterial enzyme enolase, resulting in reduced acid production by bacteria [42].

The purpose of this systematic review of the scientific literature is to compare the various remineralizing agents most widely used in clinical practice, emphasizing the advantages and disadvantages of each. What differentiates it from other literature reviews is that it has included and analyzed materials that have been most widely used in the past 5 years included in the PRISMA.

## 2. Materials and Methods

### 2.1. Protocol and Registration

The current systematic review was carried out in compliance with the standards of the Preferred Reporting Items for Systematic reviews and Meta-Analyses (PRISMA) and the International Prospective Register of Systematic Review (PROSPERO) guidelines (ID 404428).

### 2.2. Search Processing

Pubmed, Scopus, and Web of Science were searched to find papers that matched our topic, dating from 1 January 2018 up to 14 February 2023. The search method was developed by combining phrases that suited the goal of our review, which deals with the analysis of the advantages and disadvantages of white spot lesion remineralization techniques.

Hence, the following Boolean keywords were used: “enamel” AND “remineral*” AND “technique”.

### 2.3. Inclusion Criteria

The articles were selected using the following inclusion criteria: (1) studies only on human subjects; (2) open access studies; (3) clinical trials and randomized controlled trials; and (4) English language.

### 2.4. Data Processing

Two reviewers (R.M. and M.D.S.) searched the database to extrapolate the studies and assessed their quality independently, according to selection criteria. The selected articles were downloaded in Zotero (version 6.0.15). Any discrepancies between the two authors were resolved by consulting a senior reviewer (F.I.).

## 3. Results

From the search of the records on the Scopus, PubMed, and Web of Science databases, 771 articles were found, of which 142 duplicates of these records were excluded from the search before the screening.

**Figure 4:**
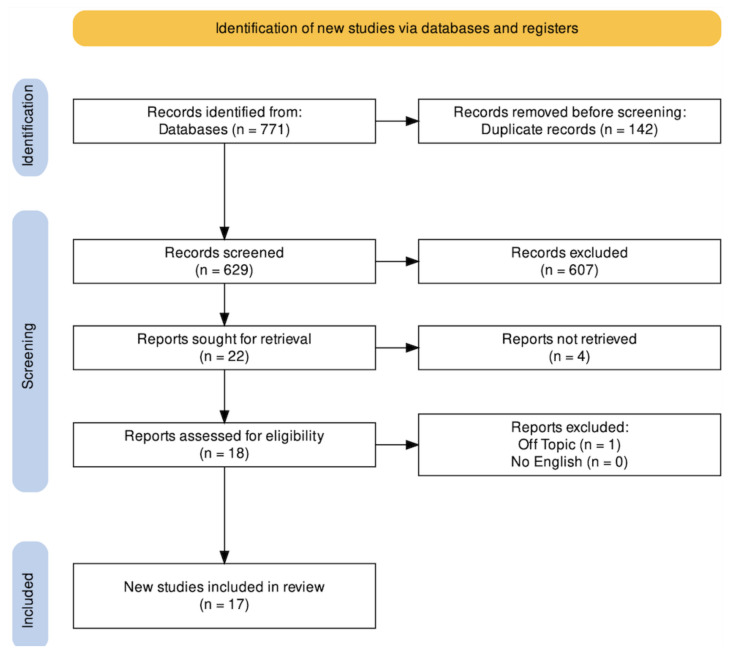
PRISMA flowchart diagram of the inclusion process. The literature search Preferred Reporting Items for Systematic Reviews and Meta-Analyses (PRISMA) flow diagram.

Hence, 629 items were reviewed and 607 items were excluded, because they were excluded from our materials and methods. Twenty-two reports were sought for retrieval and 4 reports were not retrieved. Eighteen reports were assessed for eligibility and 1 report was excluded because it was off-topic. A final number of 17 studies were included in the review for qualitative analysis (Figure 4) (Table 1).

## 4. Discussion

WSLs are the first clinical sign that can be appreciated before caries evolve into cavitated lesions. Individuals most susceptible to the occurrence of WSLs include patients undergoing orthodontic treatment. These patients tend to accumulate more plaque as a result of the presence of orthodontic accessories (brackets, elastic, and metal binding) that make oral hygiene maneuvers difficult. In both orthodontic and non-orthodontic patients, the best prevention is proper home oral hygiene instruction and motivation. This, however, is very often not enough, and therefore remineralizing agents are introduced to prevent or treat the occurrence of white spot lesions. Various remineralizing agents that can complement oral hygiene include fluoride applications (in the form of gels, varnishes, or creams) either in the office or at home. Another option may be the application of remineralizing agents independent of patient compliance, including glass ionomer composites and dental sealants [27].

Different remineralizing agents based on casein phosphopeptide, tricalcium phosphate fluoride, self-assembling peptide p11-4, nano-hydroxyapatite, ozone, fluoride, and sealants agents were compared in this systematic review.

### 4.1. Casein-Phosphopeptide-Based Studies

The most widely used and well-known products in clinical practice for WSL remineralization include both fluoride-based products and technologies based on casein-phosphopeptide-stabilized amorphous calcium phosphate complexes (CPP-CP) [30].

CPP-ACP is a casein bioactive substance that can regulate supersaturated calcium and phosphorus ions in the oral environment. When the pH of the oral cavity falls, phosphorus ions stabilize the pH, while calcium ions induce the remineralization process [60].

Among the main capabilities of CPP-ACP is to increase the permeability of fluoride ions. Therefore, the ability of fluoride ions to reach deeper lesions allows for better remineralizing effects to be achieved [61].

There are few studies in the literature investigating the efficacy of CPP-ACP topical cream used as a toothpaste compared to traditional fluoride toothpaste. However, the study by Al-Batayneh et al. found that the combination of GC Tooth Mousse and toothpaste with 500 ppm fluoride provided no greater benefit than the isolated use of the two agents [43].

The use of MI Paste Plus (casein phosphopeptide-amorphous calcium fluoride phosphate-based agent, CPP-ACFP) also made no real improvement in WSLs in post-orthodontic patients after 1 year of use [53].

Hamdi et al. concluded that an application of CPP-ACP and tricalcium silicate (TCS)-based agents twice daily achieves satisfactory remineralizing effects [52].

In the current study with these agents, the area of WSLs significantly decreased in the MI Paste Plus (65%) and Remin Pro (60%) groups over the course of the experiment, whereas there was no distinguishable improvement in the WSL area in the control group between the T2–T3 and T3–T4 time points (7% reduction over the study period). This finding suggests that MI Paste Plus and Remin Pro can successfully minimize the degree of WSLs throughout a three-month treatment period by remineralizing enamel caries.

It is thought that MI Paste Plus can keep the surface of the enamel saturated with calcium and phosphate. Moreover, MI Paste Plus’s fluoride component enhances the remineralizing capability of CPP-ACP by working in concert with it [48,55,56,62,63].

Rechmann et al. used the MIV and MIPP agents to assess CPP-ACP’s impact on WSL reversal and prevention during fixed appliance orthodontic therapy. The prescribed treatments were the use of an OTC toothpaste with 1100 ppm fluoride twice a day, daily use of MIPP, and quarterly application of MIV, and they found an improvement in the remineralization of both agents, but much better with MIPP. WSLs do not appear to be reduced appreciably by daily MIPP or quarterly MIV treatments during fixed orthodontic treatment [54].

It is possible to state how it is much more difficult to control WSLs in patients who wear orthodontic appliances than in those who do not. The potential of remineralizing agents can truly be evaluated after the removal of orthodontic appliances, which will have a long-term beneficial effect on the structure and acidity of bacterial plaque. This happens precisely because of the presence of orthodontic accessories (such as ligatures, braces, or elastics) that prevent the proper application of the remineralizing agent, which compromises its effectiveness, as can be seen in the study by Rechmann et al. [54].

Reviewing the results of the above studies, it can be stated that calcium-phosphate-based remineralization technology has proven to be a promising adjunctive treatment to fluoride therapy in the management of early carious lesions. This is possible because CPP-ACP-based remineralization technology allows high concentrations of calcium and phosphate ions to be stabilized by CPP and fluoride ions. In addition, since CPP-ACP can bind to the biofilm, it allows the pH of the plaque itself to be buffered by exerting the remineralizing effect.

### 4.2. Tricalcium Phosphate Studies

Another viable alternative to using fluoride is tricalcium phosphate (TCP) contained within ClinPro toothpaste. This agent allows for a greater penetration of fluoride ions. In particular, ClinPro 5000 (with 1.1% Sodium Fluoride) has been shown to achieve better effects than ClinPro Tooth Crème with 0.21% sodium fluoride. The use of ClinPro Tooth Crème with 0.21% sodium fluoride was more effective than ClinPro 5000, precisely because of the higher amount of sodium fluoride content [45].

The use of ClinPro Tooth Crème (0.21% NaF toothpaste with 950 ppm fluoro and f-TCP) achieves greater remineralizing results than MI Varnish with RECALDENT (CPP-ACP); while the main advantage of the latter is that of independent compliance [47].

Comparing patients treated with ClinPro Tooth Crème with a control group, AlFeel et al. demonstrated its remineralizing potential in treating WSLs. This split-mouth design is not an appropriate design for leachable materials, as described by Alfeel, 2021. If you apply Clinpro tooth creme on one side of the mouth, saliva, and the tongue can carry it to the other side of the mouth, so the result is biased and of low strength. However, despite the use of Clinpro Tooth Crème, no improvements in the aesthetics of WSLs themselves were found [50]. In contrast, ClinPro Tooth Crème used as a toothpaste may have greater efficacy, probably related to the action of brushing. The latter might give the remineralizing agent a chance to penetrate deeper. It is important to remember, however, that the brushing maneuver turns out to be highly dependent on the manual skill and cooperation of the patient. For this reason, varnishes always have a higher efficacy when compared to the former.

### 4.3. Self-Assembling Peptide 11-4 Studies

The introduction of the self-assembling peptide p11-4 (SAP11-4) enabled de novo regeneration of carious enamel, as it acts as a biomimetic. It can form new hydroxyapatite in the deeper early carious lesions, just as amelogenin supports the formation of the enamel itself [64].

Notably, the new hydroxyapatite crystals generated by SAP11-4 do not have a prismatic structure, but exhibit a “fan-like” structure, as they are arranged tangentially to the matrix fibers [65].

Broseler et al. compared the efficacy between fluoride varnish (control group) and SAP11-4 (test group) in treating early carious lesions. After one year of follow-up, a significant superiority of the results obtained with SAP11-4 over fluoride varnishes was found [56].

Even in comparison with TCPF varnish, SAP11-4 peptide demonstrated greater remineralizing potential due to its ability to self-assemble into three-dimensional fibrillar scaffolds [49].

One of the main advantages of SAP11-4 is precisely its biomimetic structure, which has enabled it to achieve better results than using fluoride or tricalcium phosphate varnishes that are more easily removed from tooth surfaces.

### 4.4. Nano-Hydroxyapatite Studies

Among the novel materials capable of remineralizing tooth enamel are nano-materials. Nano-hydroxyapatite, in particular, has distinct properties, such as increased solubility, improved biocompatibility, and greater surface energy [46].

Toothpastes composed of nano-hydroxyapatite make the remineralization process possible by releasing hydroxyapatite particles at the level of enamel prisms and dentinal tubules. Because of this ability, toothpaste containing nano-hydroxyapatite is used for the treatment of WSLs [66,67].

The first study conducted in vivo confirmed the excellent performance of nano-hydroxyapatite-containing toothpaste. With a 6-month follow-up, toothpaste containing nano-hydroxyapatite reported better results than toothpaste containing only fluoride in remineralizing WSLs [44].

Nano-hydroxyapatite exerts a significantly greater remineralizing impact on early enamel lesions than the commonly used fluorides [68].

### 4.5. Ozone Therapy Studies

Another agent with high remineralizing capabilities is ozone [69,70]. Ozone has a high bactericidal function that is exploited to treat initial caries [71]. In addition, ozone, by dilating the dentinal tubules, increases the perfusion of remineralizing agents [72]. Despite the important remineralizing abilities of ozone, its combination with a hydroxyapatite gel has been observed to produce better effects. Ozone application alone allowed for the remineralization process to take place only in the outermost portion of the enamel [46].

As compared to nano-hydroxyapatite or ozone treatment alone, the combination of both approaches delivers the best results. To obtain the impact of nonrestorative caries therapy, the treatment techniques should be sustained for a long period.

### 4.6. Fluoride Agent Studies

Fluoride and xylitol varnishes can both mineralize non-cavitated lesions, as seen by the lesions in each group regressing after application [73]. To avoid NCLs and promote remineralization during orthodontic treatment, xylitol varnishes appear to be an alternative to fluoride varnishes. After application, fluoride varnishes created a thin layer of firm varnish that adhered to the enamel surface. However, due to the complex oral environment and movement caused by the buccal muscle, tongue, mastication, saliva wash, and oral hygiene practices, a fluoride varnish is likely to be removed quickly [55,74].

Although varnishes may be easier to apply and reapply, both MI Varnish and ProSeal sealant offer comparable levels of protection while undergoing orthodontic treatments. White spot carious lesions should be treated with CPP-ACFP varnish, which is strongly advised [58,59].

One advantage is its easy application, while a disadvantage is the action of muscles and saliva that can easily remove it from the tooth surface.

### 4.7. Sealant Agent Studies

Finally, the last approach involves the use of hydrophilic resin sealant and glass ionomer sealant materials. In contrast to glass ionomer sealants, resin sealants are very sensitive to a moist environment, which can compromise their stability over time [75]. Although glass ionomer sealants have a hydrophilic nature and allow high fluorine release, they have poor retention over time. Alsabek et al. demonstrated the superiority of resin-based sealants (particularly EmbraceTM WetBondTM) over glass ionomer sealants with a 6-month follow-up [57].

A particular advantage of these sealants is that their effectiveness does not depend on patient cooperation, and therefore the results obtained following their application can be considered more reproducible and reliable.

## 5. Conclusions

After reviewing the studies, we can conclude that CPP-ACP-based remineralizing agents remain the gold standard in clinical practice, especially when combined with fluoride-containing agents. Nevertheless, from the reviewed studies, excellent results were also obtained in treating WSLs with ozone therapy, SAP11-4, nano-hydroxyapatite, and sealants. Given the unevenness and heterogeneity of the analyzed studies, it is not possible to establish the superiority of one remineralizing agent over another.

## Figures and Tables

**Table 1:** Descriptive summary of item selection.

Authors	Type of Study	Object	Study Design and Timeline	Results
O. B. Al-Batayneh, 2019 [43]	Randomized Clinical Trial	Effects of fluoride dentifrice and GC Tooth Mousse on early caries lesions.	114 children used three different agents twice daily: fluoride dentifrice (500 ppm), CPP-ACP Crème, and fluoride dentifrice + CPP-ACP Crème. Lesions compared at baseline, 3 and 6 month after.	The use of both agents provided no additional advantage over their solo usage.
Mohammadreza Badiee, 2019 [44]	Randomized Clinical Trial	Comparison of the effects of toothpastes containing nanohydroxyapatite and fluoride on white spots in orthodontic patients.	After orthodontic treatment, 50 patients used toothpaste containing fluoride or nano-hydroxyapatite. Follow-up: 1, 3 and 6 months after.	Nanohydroxyapatite toothpaste performed better than the other containing fluoride.
Chung H. Kau, 2019 [45]	Randomized Trial	Effects of three different fluoride dentifrices on white spot lesions during orthodontic treatment.	120 patients used three types of fluoride dentifrices (Clinpro 5000, Clinpro Tooth Crème and MI-Paste Plus) twice a day for 4 months.	Clinpro 5000 performs somewhat better than the other two test pastes.
Katarzyna Grocholewicz, 2020 [46]	Randomized Clinical Trial	Effect of nano-hydroxyapatite and ozone on approximal initial caries.	Comparison of three methods (a nano-hydroxyapatite gel, gaseous ozone therapy, combination of the previous two) for enamel remineralization in 92 patients with initial approximal lesions. Follow-up: 1, 2 years after.	The combination of both methods produced the best effects.
Ashish Handa, 2022 [47]	Randomized Controlled Trial	Comparison between Clinpro Tooth Creème and MI Varnish with RECALDENT^TM^ for treatment of white spot lesions.	35 patients divided into three groups: ClinproTM Tooth Creème group, Fluoride varnish group, home-care group (control).	Clinpro Tooth Crème outperforms MI Varnish with RECALDENTTM (CPP-ACP) in terms of enamel decalcification protection.
Farzin Heravi, 2018 [48]	Randomized Clinical Trial	Comparison between MI Paste Plus and Remin Pro on the remineralization of white spot lesions in postorthodontic patients.	39 patients with white spot lesions divided into three treatment groups: MI Paste Plus group, Premin Pro group, control group. Follow-up: 4, 8 and 12 weeks later.	The use of both agents helped reduce post-orthodontic white spot lesions.
Riham Kobeissi, 2020 [49]	Randomized Clinical Trial	Compare the efficacy of SAP11-4 vs. tricalcium phosphate fluoride (TCPF) in remineralizing WSLs in young permanent teeth.	Nine patients received either TCPF (group 1) or SAP11-4 (group 2). Follow-up: baseline, 3 and 6 months later.	Greater effectiveness of SAP11-4 compared with TCPF in treating white spots.
AlFeel, 2021 [50]	Randomized Controlled Clinical Trial	Effect of Clinpro Tooth Crème on remineralization of white spot lesions.	Split-mouth study: 18 patients applied Clinpro Tooth Crème on one side and no treatment on the other side.	Clinpro tooth crème has a remineralizing action on white spot lesions compared to normal oral hygiene.
Aboulnaga, 2022 [51]	Randomized Clinical Trial	Effect of Remin Pro and Remin Pro Forte on white spot lesions in orthodontic patients.	20 patients divided into two groups (Remin pro group and Remin pro forte group) were followed for 3 months.	The use of Remin Pro Forte provided greater benefits than the use of Remin Pro.
Hamdi, 2022 [52]	Randomized Clinical trial	Remineralizing potential of experimental tricalcium silicate paste (TCS) in comparison with CPP-ACP and SDF-KI.	45 patients divided into three groups (TCS, SDF-KI and CPP-ACP group). Follow-up periods: 3,6,12 and 24 months.	TCS showed potential remineralization of white spot lesions.
Beerens, 2018 [53]	Randomized Controlled trial	Remineralizing effect of MI Paste Plus (MPP) on white spot lesions after orthodontic treatments.	Long-term effect (12 months) of MPP versus a placebo paste in 65 partecipants.	The use of MPP in post-orthodontic patients did not improve the white spot lesions.
Rechmann, 2018 [54]	Randomized Controlled Trial	Effects of MI Paste Plus (MIPP) and MI Varnish (MIV) on white spot lesions in orthodontic patients.	40 patients randomly assigned to the experimental group (MIPP or MIV) or the control group.	No significant difference between MIPP and MIV.
Silva, 2021 [55]	Randomized Clinical Trial	Effects of fluoride and xylitol varnishes during orthodontic treatment.	Fluoride, xylitol varnish or placebo were applied in 55 orthodontic patients. Follow-up: baseline and 6 months after.	In short term, both varnishes produce remineralization.
Broseler, 2020 [56]	Randomized Clinical Trial	Efficacy of self-assembling peptide P11-4 and fluoride varnish in white spot lesions.	37 subject treated with P11-4 (test group) or fluoride varnish (control group). Follow-up: 1 year.	Early carious lesions treated with P11-4 were reduced.
Alsabek, 2019 [57]	Randomized Controlled Clinical Trial	Remineralization effect of resin-based sealant and glass ionomer sealent on non-cavitated pit and fissure caries.	Split mouse study on 40 patients: moisture tolerant sealant was applied on one side of the mouth and glass ionomer sealant was applied on the other side.	Both agents have demonstrated remineralizing capacity of pit and fissure caries.
Flynn, 2022 [58]	Randomized Clinical Trial	Efficacy of fluoride varnish vs. a filled resin sealant for preventing white spot lesions in orthodontic patients.	40 orthodontic patients divided into two groups: sealent group (application every 3 months); MI Varnish group (application every 4-6 weeks). Follow up: 12 months.	Similar levels of protection by both agents.
Baafif, 2020 [59]	Comparative Study	Efficacy of ICON vs. CPP-ACFP.	Split-mouth technique: 30 patients have been treated with ICON on the left side and with CPP-ACFP on the right side.	The efficacy of CPP-ACFP was better than ICON.

## Data Availability

Not applicable.

## Abbreviations

CPP-ACFP	Casein phosphopeptide amorphous calcium fluoride phosphate
HA	Hydroxyapatite
ICON	Caries infiltrant
MIPP	MI Paste Plus
MIV	MI Varnish
SAP11-4	Self-assembling peptide p11-4
TCPF	Fluoride varnish with therapeutic tricalcium phosphate formulas
TCS	Tricalcium silicate paste
WSL	White spot lesion
WSLs	White spot lesions
